# New Insights into Neutrophil Extracellular Traps: Mechanisms of Formation and Role in Inflammation

**DOI:** 10.3389/fimmu.2016.00302

**Published:** 2016-08-12

**Authors:** Hang Yang, Mona Helena Biermann, Jan Markus Brauner, Yi Liu, Yi Zhao, Martin Herrmann

**Affiliations:** ^1^Department of Rheumatology and Immunology, West China Hospital, Sichuan University, Chengdu, Sichuan, China; ^2^Department of Internal Medicine 3, Rheumatology and Immunology, Friedrich-Alexander-University Erlangen-Nürnberg (FAU), Erlangen, Germany

**Keywords:** NETosis, NETs, antimicrobial activity, autoimmune diseases, mitochondrial DNA

## Abstract

Recent data suggest that NETosis plays a crucial role in the innate immune response and disturbs the homeostasis of the immune system. NETosis is a form of neutrophil-specific cell death characterized by the release of large web-like structures referred to as neutrophil extracellular traps (NETs). NETs are composed of DNA strands associated with histones and decorated with about 20 different proteins, including neutrophil elastase, myeloperoxidase, cathepsin G, proteinase 3, high mobility group protein B1, and LL37. Reportedly, NETosis can be induced by several microbes, and particulate matter including sterile stimuli, *via* distinct cellular mechanisms. Meanwhile, suicidal NETosis and vital NETosis are controversial. As we enter the second decade of research on NETosis, we have partly understood NETs as double-edged swords of innate immunity. In this review, we will discuss the mechanisms of NETosis, its antimicrobial action, and role in autoimmune diseases, as well as the relatively new field of NET-associated mitochondrial DNA.

## Introduction

Neutrophil granulocytes are the most abundant type of white blood cells in humans and play a vital role in innate immunity by defending the host against invading pathogens. The immune regulatory functions of neutrophils include phagocytosis, generation of reactive oxygen species (ROS), degranulation, and the formation of neutrophil extracellular traps (NETs), a process referred to as NETosis. NETosis is accepted as a specific form of cell death subroutine performed by granulocytes, differing from apoptosis and necrosis (1, 2). When neutrophils undergo NETosis, nuclear and granular membranes disintegrate, the chromatin decondenses, and it diffuses into the cytoplasm, mixing with cytoplasmic proteins. This is followed by plasma membrane rupture and the release of chromatin, decorated with granular proteins, into the extracellular space (2, 3). NETs consist of chromatin fibers with diameters of 15–17 nm that contain DNA and the histones H1, H2A, H2B, H3, and H4. Moreover, the DNA fibers are decorated with several proteins like neutrophil elastase (NE), myeloperoxidase (MPO), cathepsin G, proteinase 3 (PR3), high mobility group protein B1 (HMGB1), and LL37, thus displaying proinflammatory characteristics (1). In the past decade, new aspects of neutrophil functions have emerged unveiling their significance not only in defending the host against microbes but also in contributing to many autoimmune pathological conditions. Therefore, the purpose of this review is to present and discuss the current knowledge about the mechanisms of NETosis and its role in the pathogenesis of autoimmune diseases.

## Mechanisms of NETosis

Neutrophil suicide, distinct from either necrosis or apoptosis, was first described following chemical stimulation with phorbol 12-myristate 13-acetate (PMA) in 1996 (4). This form of cell death was characterized by the disintegration of nuclear and granular membranes and by the release of decondensed chromatin into the cytoplasm. In 2004, Zychlinsky and colleagues reported that neutrophil suicide resulted in the release of large web-like structures composed of decondensed chromatin and neutrophil antimicrobial factors, and coined the name neutrophil extracellular traps (1). In their studies, they used PMA and interleukin-8 (IL-8) to elicit NETs *in vitro*. In 2007, it was reported that, going along with chromatin decondensation, neutrophils undergo an NADPH oxidase-dependent death process that includes nuclear envelope disintegration and the mixing of nucleic acids and granule proteins within a large intracellular vacuole (3). After the association of nucleic acids and granule proteins, NETs are released *via* plasma membrane perforation and cell lysis. This process is completed1–4 h after the inciting stimulus. The released chromatin structures are prone to bind particular matter, e.g., bacteria. The authors concluded that PMA-induced NETosis is a form of a beneficial suicide (3). Apart from PMA and IL-8, bacteria, fungi, protozoa, antibody–antigen complexes (5), autoantibodies (6), tumor necrosis factor (TNF), interferon (IFN) (7), and further stimuli also trigger NETosis.

## Pathways

Conventional suicidal NETosis has long been recognized as a distinct form of active cell death. In addition, some researchers have described a different mechanism by which NETs are formed, termed vital NETosis. This non-suicidal pathway allows NET release from neutrophils staying viable (8–12).

## Conventional Suicidal NETosis

**Figure 1:**
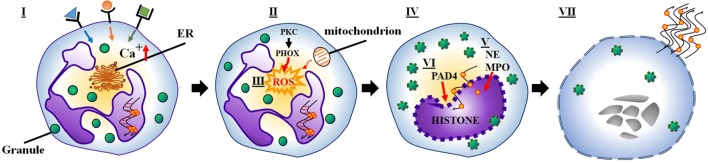
**(I) Several stimuli (e.g., bacteria, viruses, fungi) initiate NETosis by binding to neutrophil receptors (e.g., Fc receptors, TLRs), which activate the endoplasmic reticulum to release stored calcium ions**. (II) Elevated cytoplasmic calcium levels increase PKC activity, which induces NADPH oxidase to assemble into a functional complex (PHOX). (III) Subsequently, PHOX (or alternatively the mitochondrial respiratory chain) generate ROS. (IV) ROS generation leads to the rupture of granules and the nuclear envelope. (V) Meanwhile, NE and MPO translocate to the nucleus. (VI) As a result, histone deimination and chromatin decondensation contribute to the formation of NETs. (VII) Finally, the rupture of the plasma membrane causes neutrophil lysis and allows the release of NETs.

Conventional suicidal NETosis is frequently initiated by ligand binding to neutrophil toll-like receptors and receptors for IgG–Fc, complement, or cytokines (1, 5, 13). Upon activation of these receptors, calcium storages of the endoplasmic reticulum release calcium ions into the cytoplasm. Elevated cytoplasmic calcium levels increase protein kinase C (PKC) activity and phosphorylation of gp91phox (14). This induces the assembly of the cytosolic and membrane-bound subunits of NADPH oxidase into functional complexes at cytoplasmic or phagosomal membranes (also called phagocytic oxidase, PHOX) and the subsequent generation of ROS (15). Under the influence of ROS, granules and the nuclear envelope rupture. Subsequently, the released nuclear, granular, and cytoplasmic contents blend. NE and MPO, usually stored in azurophilic granules, migrate to the nucleus. Here, NE degrades the linker histone H1 and processes the core histones, and MPO enhances chromatin decondensation (15). Histone deimination by peptidyl arginine deiminase 4 (PAD4) and proteolytic cleavage of histones initiated before nuclear breakdown additionally contribute to chromatin decondensation (16, 17). The rupture of the plasma membrane allows the release of NETs and leads to cell death and the loss of viable cell functions of like migration and phagocytosis (Figure 1) (15).

## Reactive Oxygen Species

The generally accepted notion that ROS play a crucial role in the classical suicidal NETosis pathway is based on two important observations: (1) Neutrophils from patients with chronic granulomatous disease (CGD), not capable of performing the oxidative burst, show strongly reduced abilities to form NETs. This is independent of the type of mutation leading to a defective PHOX complex. CGD patients suffer from severe and often chronic infections (3, 18). Moreover, treatment with H_2_O_2_ rescued the production of NETs in neutrophils from CGD patients, downstream of the PHOX complex (3). (2) ROS scavengers, such as *N*-acetylcysteine, or trolox reportedly inhibit NETosis (3, 19). In fact, it remains unclear how ROS participate in the dismantling of the nuclear envelope or the mixing of the NET components. Some studies suggest that ROS directly promote the morphologic changes observed during NETosis (14). ROS may alternatively inactivate caspases, thereby inhibiting apoptosis and favoring autophagy. This leads to dissolution of cellular membranes (20). These two alternatives are not mutually exclusive: under certain experimental conditions, each of them can also act independently. There is now growing evidence that some stimuli induce NETosis independent of NADPH oxidase. Oxidant-independent release of NETs was studied in detail by Winterbourn and colleagues (21).

## Peptidyl Arginine Deiminase 4

Peptidyl arginine deiminase 4 catalyzes the conversion of arginine residues to citrullinein polypeptides, thereby eliminating a positive charge of the protein. Thus, citrullination of histones weakens the stability of nucleosomes (22, 23). Loss of positive charges causes the opening of the compact structure of chromatin and allows decondensation and dispersion of chromatin in the form of NETs. Consistently, neutrophils from mice with a PAD4 deficiency display impaired capacities to form NETs and are highly susceptible to severe skin infections *in vivo* (16, 17). However, PAD4 deficiency does not contribute to lung infections caused by influenza virus (16).

## Vital NETosis

**Figure 2:**
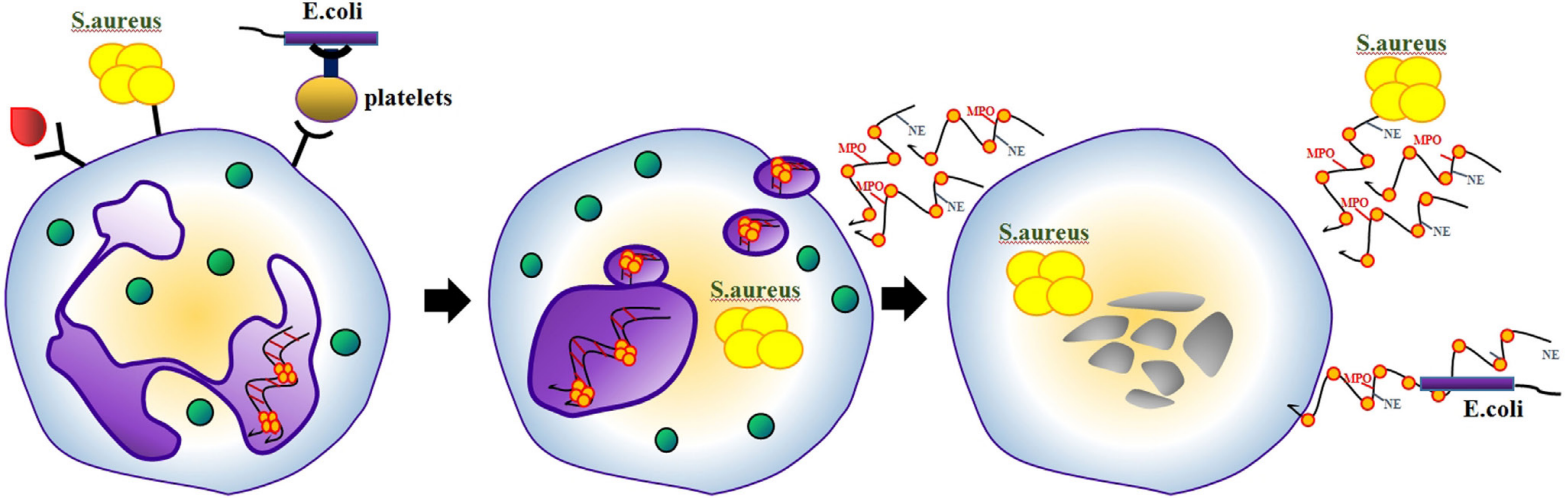
**Some NETosis-inducing stimuli involve TLR4 on platelets**. Under these conditions, neutrophils release NETs *via* blebbing of the nuclear envelope and vesicular exportation. As a result, neutrophils become nuclear cytoplasts, which are still able to migrate and retain several conventional functions of viable neutrophil.

Contrary to previous studies describing the canonical pathways of NETosis as a process requiring several hours, Clark et al. reported in 2007 that lipopolysaccharide (LPS)-stimulated NETosis occurred within just 30 min involving TLR4 on platelets (8). It was demonstrated that neutrophils that released NETs remained impermeable for SYTOX Green, indicating that they remained structurally intact. Therefore, the authors later coined the term vital NETosis (12). Electron microscopy revealed that NET release induced by *Staphylococcus aureus* occurs *via* blebbing of the nuclear envelope and vesicular exportation *in vitro* and *in vivo* (9). As a result, this pathway preserved the integrity of the neutrophils’ plasma membranes (Figure 2). NETting neutrophils became anuclear cytoplasts capable of chasing and imprisoning live Staphylococci (10). *Candida albicans* was reported a further stimulus of vital NETosis (11). It still remains controversial whether and how suicidal and vital NETosis coexist. Furthermore, it is not clear if a neutrophil that has ejected (parts of) its DNA should be termed “viable.”

## Mitochondrial DNA

As mentioned above, ROS are indispensable for several kinds of NETosis (24). In mammals, both the mitochondrial respiration chain and the NADPH oxidase independently contribute to the production of ROS (25). Recently, it has been observed that *in vivo* inhibition of mitochondrial ROS production reduced intracellular ROS levels and NETosis (26). Ribonucleoprotein immune complexes (RNP ICs) were used to stimulate neutrophils and mitochondrial ROS generation. Mitochondria became hypopolarized, translocated to the cell surface, and were observed within the expelled NETs. Concomitantly, mitochondrial ROS oxidized mitochondrial DNA (mtDNA), which is proinflammatory *in vitro*. When injected into mice, oxidized mtDNA triggered inflammation and type I IFN production *via* a pathway dependent on the DNA sensor STING (7, 26). Mitochondria have evolved from bacteria and contain unmethylated CpG motifs (27) as well as *N*-formylated peptides (28). Similar to bacteria, extracellular mitochondria are stimulators of proinflammatory signaling. Several reports attribute this effect to the unmethylated CpG DNA repeats within the mtDNA (29), others highlight the effect of DNA oxidation (28).

In patients with systemic lupus erythematosus (SLE), abnormal NETosis and defects in the clearance of NETs were found to promote the production and release of type I IFN (30). In contrast, patients with CGD carry an increased risk to suffer from SLE, despite lacking functional NADPH oxidase activity (18), the major source of ROS in activated healthy neutrophils. Based on this observation, one might question whether increased NETosis is a factor contributing to the etiopathogenesis of SLE. Instead, deficiency of the clearance of NETs is likely to foster the antinuclear autoimmunity in patients with SLE (30, 31). However, Kaplan and colleagues reexamined the importance of ROS in low-density granulocytes from patients with CGD and observed that this granulocyte subpopulation undergoes spontaneous NETosis and that their mitochondrial respiration produces sufficient amounts of ROS to execute NETosis. The levels of ROS derived from low-density granulocytes correlated with the levels of type I IFN in the corresponding patients. Accordingly, the authors not only confirmed that mitochondria drove NETosis but also concluded that NETosis is a pathological factor able to foster SLE (26, 32). Consistently, anti-mtDNA antibodies were elevated in the sera of patients with SLE, and antibody levels correlated with IFN scores and disease activity. Immune complexes containing mtDNA induced more IFN-α than those with nuclear dsDNA. Thus, anti-mtDNA antibodies can be considered as typical for driving both SLE and lupus nephritis (33). As a drug that selectivity inhibits mitochondrial respiratory chain complex I and decreases NADPH oxidase activity (34), metformin may be a new option to treat SLE (33).

## Antimicrobials

Numerous microbes reportedly induce formation of NETs (Table 1). NET-inducing molecules include the bacterial cell surface components LPS, lipoteichoic acid, and their breakdown products. Several bacteria and fungi were reported to potently induce NET formation, such as *Staphylococcus aureus* (9, 35), *Streptococcus* sp. (36), *Haemophilus influenzae* (37), *Klebsiella pneumonia* (15), *Listeria monocytogenes* (38), *Mycobacterium tuberculosis* (39), *Shigella flexneri* (1), *Aspergillus nidulans, Aspergillus fumigatus*, and *Candida albicans* (40–42). Further examples are pathogens, such as *Yersinia* (1) and members of the oral microbiome, including *Porphyromonas gingivalis* (43). NETs can immobilize and kill a broad range of microbes, including bacteria, fungi, and protozoa (1, 9, 15, 35–42), and thus prevent the dissemination of microbial pathogens (37). Some studies have questioned the killing capabilities of NETs since viable *Staphylococcus aureus* and *Candida albicans* blastospores were released from NETs by incubation with DNases (44). Branzk et al. found that in response to large pathogens, like filamentous *Candida albicans*, neutrophils selectively released NETs (45). Intriguingly, NETosis did not occur in response to the yeast form of *Candida albicans* or single bacteria. Phagocytosis *via* dectin-1 acted as a sensor of microbial size and prevented NET release by downregulating translocation of NE to the nucleus. Apart from directly killing microbes, NETs inactivate microbial “virulence factors” that alter the function of host cells. NET-associated NE specifically cleaved virulence factors of *Shigella flexneri, Salmonella typhimurium*, and *Yersinia enterocolitica* (1). The serine proteases cathepsin G and PR3 may also destroy virulence factors of further classes of microbes (46). NETs contain several proteins that inhibit microbes, including enzymes, antimicrobial peptides, calgranulin, and histones. The microbicidal activity of NETs results from the combined action of several components being enhanced by the high local concentrations of mediators on the NETs’ surfaces (15).

**Table 1:** **Pathogens that induce NETs**.

Species	Reference
*Staphylococcus aureus*	(9, 35)
*Streptococcus* sp.	(36)
*Haemophilus influenzae*	(37)
*Klebsiella pneumoniae*	(15)
*Listeria monocytogenes*	(38)
*Mycobacterium tuberculosis*	(39)
*Shigella flexneri*	(1)
*Aspergillus nidulans*	(40)
*Aspergillus fumigatus*	(41)
*Candida albicans*	(42)
*Yersinia*	(1)
*Porphyromonas gingivalis*	(43)
*V. cholera*	(51)
*Aeromonas hydrophila*	(58)

Various components of NETs contribute to different aspects of microbicidal activity. It was shown that the activity of MPO on NETs is essential to eliminate *S. aureus* (47). The antifungal activity of NETs has been assigned to calgranulin (48), which chelates zinc, a cation required for fungal growth (15). Also, histones restrict microbial growth very efficiently, and antibodies against histones prevent NET-mediated microbicidal activity (1). Microbes are suggested to be entrapped due to electrostatic interactions between the positively charged bacterial surface and the negatively charged chromatin fibers based on electrostatic interactions (49). Encapsulated pathogens or those that can change their surface charge may escape entrapment (50). Importantly, several bacteria are able to degrade NETs by nucleases and thus escape NET-mediated entrapment (Table 2). These include the Gram-negative pathogen *Vibrium cholera* (51) and the Gram-positive bacteria *Streptococcus pneumoniae* (52), *Streptococcus pyogenes* (53), *Yersinia* ssp. (54), *Streptococcus agalactiae* (55), *Streptococcus suis* (56), *Staphylococcus aureus* (57), and *Aeromonas hydrophila* (58). This emphasizes the importance of nucleases as pathogenic factors.

**Table 2:** **Pathogens which evade entrapments *via* degrading NETs**.

Species	Reference
*V. cholera*	(51)
*Streptococcus pneumoniae*	(52)
*Streptococcus pyogenes*	(53)
*Yersinia*	(54)
*Streptococcus agalactiae*	(55)
*Streptococcus suis*	(56)
*Staphylococcus aureus*	(57)
*Aeromonas hydrophila*	(58)

## The Role of NETosis in Autoimmune Diseases

### Vasculitis

Vasculitis manifests in vessel wall inflammation and can affect any organ system of the body. ANCA-associated vasculitis (AAV), a subgroup of the vasculitides, is characterized by involvement of the small vessels, a neutrophil-rich necrotizing inflammation, and the presence of anti-neutrophil cytoplasmic antibodies (ANCAs) (59). AAV comprises granulomatosis with polyangiitis (formerly Wegener’s granulomatosis), microscopic polyangiitis, and eosinophilic granulomatosis with polyangiitis (formerly Churg–Strauss syndrome). Many ANCAs are directed against PR3 or MPO, enzymes typically found in the azurophilic granules of neutrophils and on the surfaces of NETs (60). NETs are reportedly released by ANCA-stimulated neutrophils and in turn contain the autoantigens PR3 and MPO (39). This suggests that NET formation triggers vasculitis and promotes the autoimmune response against neutrophil components in individuals with small-vessel vasculitis (61). Consistently, increased levels of NET remnants containing complexes of nucleosomes and MPO have been detected in the circulation of patients with active vasculitis (39) and in patients with active AAV (60). Neutrophils of patients with AAV exhibited an increased tendency for spontaneous cell death. The levels of NET remnants were positively correlated with disease activity and neutrophil count, but inversely with ANCA at least during remission.

### Systemic Lupus Erythematosus

Systemic lupus erythematosus is a complex multifactorial autoimmune disease associated with severe organ damage. NETs are considered a potential source of autoantigens. Polymorphonuclear leukocytes (PMNs) of patients with SLE display an increased propensity to execute NETosis in conjunction with impaired degradation of NETs by circulating DNase1. The aberrant NETs induce type I IFN, which is associated with vascular complications and tissue damage (30, 62).

High numbers of low-density granulocytes have been identified as a particular subset of neutrophils in SLE patients. Low-density granulocytes persistently produced TNF and type 1 IFN, and spontaneously underwent NETosis (24). Furthermore, increased IFN-α in SLE patients is an important driving force that primes neutrophils for the execution of NETosis (63).

Not only production but also degradation of NETs is altered in SLE patients. Sera of a subgroup of SLE patients degrade NETs less efficiently than those of healthy controls (30). The deficient clearance of NETs in patients with SLE correlates with high titers of anti-NET antibodies and renal involvement (30). In healthy individuals, mononuclear phagocytes clear NETs in cooperation with DNase1 and C1q both synergizing in predigesting the chromatin part (64). The activities of serum DNase1 in patients with SLE are lower than that of healthy controls (65). Increased serum levels of DNase1 inhibitors, rare mutations in the gene of DNase1, and anti-DNase 1 antibodies may explain the decreased activity of DNase 1 (66, 67). Circulating chromatin in the form of immune complexes in individuals with SLE contains LL37, which triggers TLR9 in plasmacytoid dendritic cells, induces IFN-α synthesis, and protects nucleic acids from degradation by nucleases (68, 69). A study found that the individual NET degradation activity in the circulation of a given patient changed with disease activity. Sera of patients with SLE, which were not able to degrade NETs, showed increased complement consumption, since NETs activate the classical complement pathway due to their interaction with C1q (70). Thus, strategies that eliminate NETs and their components from the circulation pose a promising therapeutic approach for the treatment of patients with SLE (70).

### Thrombosis

Neutrophil extracellular traps promote thrombosis by providing a scaffold and stimulus for platelet and red blood cell adhesion and aggregation (71), thus enhancing coagulation (72). Neutrophils in thrombi are required for propagation of deep venous thromboses by binding factor XII and supporting its activation through NETosis (73). The major components of NETs (DNA, histones, and proteases) all display procoagulant properties. DNA induces thrombin generation in plasma and increases the protease activity of coagulation factors (74, 75). Histones may directly induce epithelial and endothelial cell death (76), and can mediate thrombosis *in vivo* (77). Histones were found to inhibit anticoagulation of plasma by promoting thrombin generation and hamper thrombomodulin function (78, 79). Elastase inactivated the tissue factor pathway inhibitor; thus, further increasing coagulation and fibrin deposition *in vivo* (72). Release of NETs in the vascular compartment triggered a procoagulant state and promoted binding and activation of platelets leading to thrombosis (80).

### Rheumatoid Arthritis

In the autoimmune disease, rheumatoid arthritis (RA), the formation of autoantibodies to citrullinated proteins (ACPA) is thought to be a key pathogenic factor. Given that histone citrullination is implicated in NET formation, NETosis may play a critical role in RA (81). In 2013, Kaplan and colleagues found that neutrophils from patients with RA had a greater tendency to release NETs than neutrophils from healthy controls. RA serum and synovial fluid was a strong inducer of NETosis (82). Furthermore, NETosis resulted in the externalization of citrullinated protein antigens and immune-stimulatory molecules that may promote aberrant adaptive and innate immune responses in the joint.

### Diabetes

Diabetes mellitus (or diabetes) is a chronic, lifelong condition, in which impaired insulin secretion and variable degrees of peripheral insulin resistance lead to hyperglycemia and affect the body’s ability to use food energy. Under conditions of hyperglycemia, neutrophils reportedly produce more superoxide and cytokines, like TNF-α, which triggers NETosis (83, 84). Based on these studies, we speculated that hyperglycemia may facilitate NETosis. Recently, Wong et al. (85) isolated neutrophils from type 1 and type 2 diabetic humans and mice. Nearly twice as many neutrophils derived from patients released NETs in comparison to cells from healthy controls. The authors attributed this to PAD4 and revealed a fourfold upregulation of PAD4 protein expression in the neutrophils from individuals with diabetes as compared to healthy controls. It is well established that delayed wound healing is a hallmark of patients with diabetes. The authors reported that large quantities of NETs were found in excisional skin wounds of diabetic mice and that DNase1, which dismantled NETs, accelerated wound healing. Despite the triggers of NETosis in wounds remaining elusive, it has been confirmed that inhibiting NETosis or degrading NETs improved wound healing and reduced NET-driven chronic inflammation in diabetes (85). However, the exact role of NETosis in wound healing remains to be revealed.

### Cancer

NETosis may influence tumor development during many stages, including growth, angiogenesis, and metastasis. It has been observed that there is a large necrotic area of dead neutrophils and NET-like structures in Lewis lung carcinoma and Ewing sarcoma (86, 87). It remains to be clarified whether these NETs are responsible for the generation of the necrotic areas. Alternatively, NETs may serve to shield healthy tissues from necrotic areas. A study observed NET deposition on the microvasculature and subsequent local trapping of circulating cancer cells. The tumor cells, immobilized by NETs, survived and proliferated to form nodules. This suggests a role for NETs in enhancing tumor metastasis (88). However, whether NETs just protect or anchor cancer cells physically or whether they promote tumor growth is still elusive.

### Sepsis

The pathology of sepsis results from infection, hyperinflammatory host response, and immune paralysis. During sepsis, NETs are released in the vascular system, where they trap bacteria (8, 12). Trapped bacteria can be killed, protecting patients from bacterial overflow (1, 44). In contrast, NET deposition in organs and their pro-thrombotic activities may also contribute to organ failure (89, 90). When researchers subjected mice to polymicrobial sepsis following cecal ligation and puncture, PAD4-deficient mice showed a similar survival rate when compared to wild-type controls (91). However, PAD4-deficient mice were partially protected from LPS-induced shock, indicating that NETs may contribute to the toxic inflammatory and procoagulant host response to bacteria in sepsis. The authors proposed that preventing NET formation by PAD4 inhibition in inflammatory or thrombotic diseases is not likely to increase host vulnerability to bacterial infections (91).

## Conclusion

Progress in the research on NETosis has greatly increased our understanding of its role in immunological processes and autoimmune disorders. Recent studies described how autoantigens, released during NETosis, activate immune cells and that cytokines in turn give rise to further NETosis. Aggregated NETs finally sequester and degrade proinflammatory mediators to avoid excessive inflammation (8, 14). The published data also revealed that blocking the process of NETosis or inhibiting the activity of components in NETs might be effective in the treatment of autoimmune diseases. Future work investigating the exact process of NETosis and the interplay of NET components and the immune system will contribute to a deeper understanding of the role of neutrophils in the induction and resolution of inflammation.

## Author Contributions

HY and YZ wrote the first draft of this article. HY and YL designed the figures. YL, MB, JB, and MH critically revised the manuscript for important intellectual content. All authors approved the final version.

## Conflict of Interest Statement

The authors declare that the research was conducted in the absence of any commercial or financial relationships that could be construed as a potential conflict of interest.
